# Variation in gestational diabetes diagnosis and care practices in maternity services in three high-income countries; a cross-sectional survey

**DOI:** 10.1186/s12884-025-08472-5

**Published:** 2025-12-06

**Authors:** Anna Davies, Amy Howell, Sharleen O’Reilly, Helena Teede, Cheryce Harrison, Gemma Clayton, Fionnuala M. McAuliffe, Aisling Geraghty, Charlotte Williams, Christy Burden

**Affiliations:** 1 Academic Women’s Health Unit, Bristol Medical School, University of Bristol, Bristol, UK; 2 School of Agriculture and Food Science, University College Dublin, Dublin, Ireland; 3 Monash Centre for Health Research and Implementation, Monash Health and Monash University, Melbourne, Australia; 4 Population Health Sciences, Bristol Medical School, University of Bristol, Bristol, UK; 5 MRC Integrative Epidemiology Unit, University of Bristol, Bristol, UK; 6 UCD Perinatal Research Centre, University College Dublin, National Maternity Hospital, Dublin, Ireland; 7 North Bristol NHS Trust, Bristol, UK

**Keywords:** Gestational diabetes, Cross-Sectional studies, Practice guidelines, Diagnostic tests, Risk factors

## Abstract

**Background:**

There is limited international consensus about best diagnostic and care practices for gestational diabetes mellitus (GDM). This impacts consistency of care and perinatal outcomes. We aimed to explore GDM diagnostic and care practices in the UK, Ireland and Australia to better understand these variations and determine areas for improvement.

**Methods:**

We conducted a cross-sectional survey of healthcare professionals between August 2021-November 2022. The survey evaluated the use of GDM guidelines, associated diagnostic practices and post-diagnostic care. Data were descriptively analysed.

**Results:**

Sixty-three maternity centres were represented (35 England and Wales, 12 Ireland, 16 Australia). 94% of centres in England and Wales and Australia respectively used their national guidelines to select women for GDM testing, with a wider variety of guidelines used in Ireland. Over 80% of centres across countries test women for GDM based on risk-factors identified in early pregnancy. At 24–28 weeks’ gestation, 94% of Australian centres used universal screening as per national guidelines, compared with 3% of centres in England & Wales, and 50% in Ireland, where universal screening is not included in national guidelines. Risk factors used to select women for screening varied between countries and between centres within countries at both time points, with some centres using risk factors outside of their national guidelines. Diagnostic tests for GDM varied between countries and between centres within countries, and according to gestation and previous GDM. Insulin was the most common first-line treatment in Australia, whereas in Ireland and England and Wales it was Metformin. Birth was planned at differing gestations according to centre and country, and according to management strategy.

**Conclusions:**

GDM-related practices vary within and between Australia, Ireland and England and Wales. National guidelines are not adhered to in some centres, which could result in inconsistent care within countries, and could result in inequitable perinatal outcomes. Further research should address standardised, evidence-informed care and guideline implementation barriers.

**Supplementary Information:**

The online version contains supplementary material available at 10.1186/s12884-025-08472-5.

## Introduction

Gestational Diabetes Mellitus (GDM) is becoming the most common pregnancy complication, with an estimated standardised global prevalence of 14% [1]. Estimated prevalence is 8%−24% in the United Kingdom (UK) and Ireland [2] and 18% in Australia [3, 4]. Prevalence is increasing due to increasing advanced maternal age and obesity, and alterations to testing and diagnostic criteria [2, 4]. GDM is associated with adverse pregnancy outcomes including pre-term birth, caesarean section, shoulder dystocia, stillbirth and neonatal intensive care admission [5–7]. Long-term consequences include increased risk of type 2 diabetes (T2DM) for women [8], and cardiovascular disease risk for women and children [9, 10]. Effective diagnosis and treatment of GDM are therefore vital for improving these short- and long-term impacts.

There is a lack of international consensus regarding best practice for GDM screening, testing and diagnostic criteria. The International Association of Diabetes and Pregnancy Study Groups (IADPSG) recommends universal screening using an oral glucose tolerance test (OGTT) [11]. While Australia has adopted these guidelines [12], the UK and Ireland have their own national guidelines, which differ from IADPSG recommendations and from each other [13, 14](see supplementary file 1 for guidelines). These differences, and the differing populations in which they are applied, contribute to variations in GDM prevalence over time and between countries [15]. The UK, Ireland and Australia all offer universal or public maternity healthcare free of charge. While national and international guidelines inform care in these settings, it is recognised that implementation variations may exist due to external and internal factors [16]. Therefore, the extent to which GDM-related care is consistent within and between countries is unclear.

We aimed to explore GDM screening, diagnosis and management practices in relation to national guidelines in the UK, Ireland and Australia.

## Method

The study is reported in accordance with the CHERRIES checklist for reporting online surveys (supplementary File 2) [17]. 

### Ethics

Ethical approval was obtained from the University of Bristol Faculty of Health Sciences Research Ethics Committee (reference: 111987), aligning with the Declaration of Helsinki policy statement regarding medical research involving human participants.

### Design and setting

A cross-sectional online survey undertaken between August 2021 and November 2022, targeting maternity services in the UK, Australia and Ireland.

### Participants and recruitment

A convenience sample of maternity healthcare professionals providing GDM-related care to women in the UK, Ireland and Australia was recruited, including obstetricians, endocrinologists, diabetes specialist nurses and diabetes midwives. The open, voluntary survey was distributed via X (formerly Twitter) and email contact lists for national professional networks (UK: Macdonald Obstetric Medicine Society, Royal College of Midwives, South-West Pregnancy Diabetes Health Care Professional Group; Australia: Society for Obstetric Medicine Australia and New Zealand, Australasian Diabetes in Pregnancy Society, Australian Diabetes Educators Association; Ireland: Irish Nutrition and Dietetic Institute, Institute of Obstetricians and Gynaecologists, Royal College of Physicians). Healthcare professionals known to the study team in Ireland were directly contacted. The survey was shared 2–3 times in each network. No incentive was offered to participate.

### Patient and public involvement

Patients and the public were not involved in the design or conduct of this study since health care professionals were targeted survey participants.

### Survey

The survey was undertaken within the English speaking countries of the Impact Diabetes Bump2Baby&Me (IDB2B&Me) project, a randomised controlled trial in four countries (UK, Ireland, Australia, Spain), in which a digital intervention for women with GDM risk was evaluated in an implementation and effectiveness study [18]. The survey was collaboratively developed with country leads (CB, SOR, HT) and through scoping interviews with clinical staff in each country. It was piloted and refined with clinicians not involved in delivery of IDB2B&Me at each site before distribution.

The survey comprised eight screens with 4–24 questions per page. Respondent burden was minimised through use of multiple choice and open-ended questions and adaptive questioning, wherein some questions only displayed based on previous item responses. We did not randomise question order to ensure adequate data capture for key questions of interest.

The survey was hosted on the Research Electronic Data Capture (REDCap [19]) and Qualtrics XM [20] platforms. Information was provided before the participant consented to participate. At the start respondents completed demographic questions on role, country and maternity care facility. This was followed by core questions on guidelines used to select women for testing and diagnosis, universal versus selective testing, screening criteria applied, diagnostic tests used. The final section explored post-diagnosis care: support offered, medications used, timing of induction of labour and caesarean section. Participants could leave items blank if they lacked knowledge about the issue or did not wish to complete it and could review their responses using a ‘back’ button. There was no completion check before submission.

### Analysis

#### Data cleaning

Entries were removed if the country or hospital was unknown. Duplicate entries from the same email address were removed. Data were included if the respondent answered at least one survey question after demographics.

Individual hospitals/maternity care facilities were referred to as maternity ‘centres’. Each named hospital is treated as an individual centre within the data; while multiple maternity care facilities may be administered by a single healthcare provider, they may have differing practices due to HCP preference or relevant to their local population. Where multiple staff from the same centre responded, data were merged where possible. Differing responses within the same centre were both included. Therefore, the number of responses for some questions is greater than the total number of centres participating in the survey. Due to limited representation from Scotland (*n* = 2) and Northern Ireland (*n* = 1), these countries’ data were excluded. UK data therefore represents responses from England and Wales only.

#### Analysis

Data were analysed in RStudio version 4.2.1 [21]. Completion rates were calculated for each question (number of participants completing the question/total number of participants who started the survey). Descriptive statistics were used to explore healthcare practices within and between countries. No inferential analyses were undertaken. Data were visualised using histograms and tables. The sample size for each question is presented in the respective figure/table.

## Results

Twelve centres incorporating 38 individual responses in Ireland (63% of 19 maternity hospitals), 35 centres incorporating 45 individual responses in England and Wales (22% of NHS trusts in England, 3 of 7 Health Boards in Wales) and 16 centres incorporating 19 individual responses in Australia (8% of 230 providers) participated. Respondents’ job roles are presented in supplementary file 3. Completion of core questions ranged between 63% and 100% (supplementary file 4).

### GDM testing

In Australia and England and Wales, most centres (*n* = 15/16 Australia, *n* = 33/35 England and Wales) used their national guideline to determine who is offered GDM testing (see supplementary file 5). In Ireland, all centres used the Health and Safety Executive (HSE) guideline, however several centres also used other guidelines alongside, including NICE (*n* = 7/12; 58%), Royal Australian and New Zealand College of Obstetrics and Gynaecology (RANZCOG; *n* = 3/12; 25%)) and ‘other’ guidelines (*n* = 3/12; 25%).

*In early pregnancy (12–14 weeks gestation)*, 81–100% of centres across all three countries selected women for GDM testing based on risk factors. Contrary to HSE, NICE and RANZCOG/ADIPS guidelines (see Supplementary file 1), two of eight Irish centres (25%), 6 of 30 centres (17%) in England and Wales and 3 of 16 (19%) of Australian centres also reported universal testing (see supplementary file 6).

*At 24–28 weeks gestation*, as per RANZCOG/ADIPS guidelines 94% (*n* = 15/16) of Australian centres reported universal testing. Contrary to their national guidelines, half of Irish centres (*n* = 6/12) reported doing so. However, 44% (*n* = 7/16) of Australian centres and 92% (*n* = 11/12) of Irish centres also reported selective testing. Three-quarters of Australian (*n* = 12/16) centres and 58% (*n*=7/12) of Irish centres reported using both. All centres in England and Wales used selective testing (as per NICE guidelines), with just one centre (*n* = 1/34; 3%) indicating using both. This indication of both universal and selective testing being used may have resulted from individuals from the same centre offering differing responses.

### Risk factors used to select women for GDM testing

**Fig. 1:**
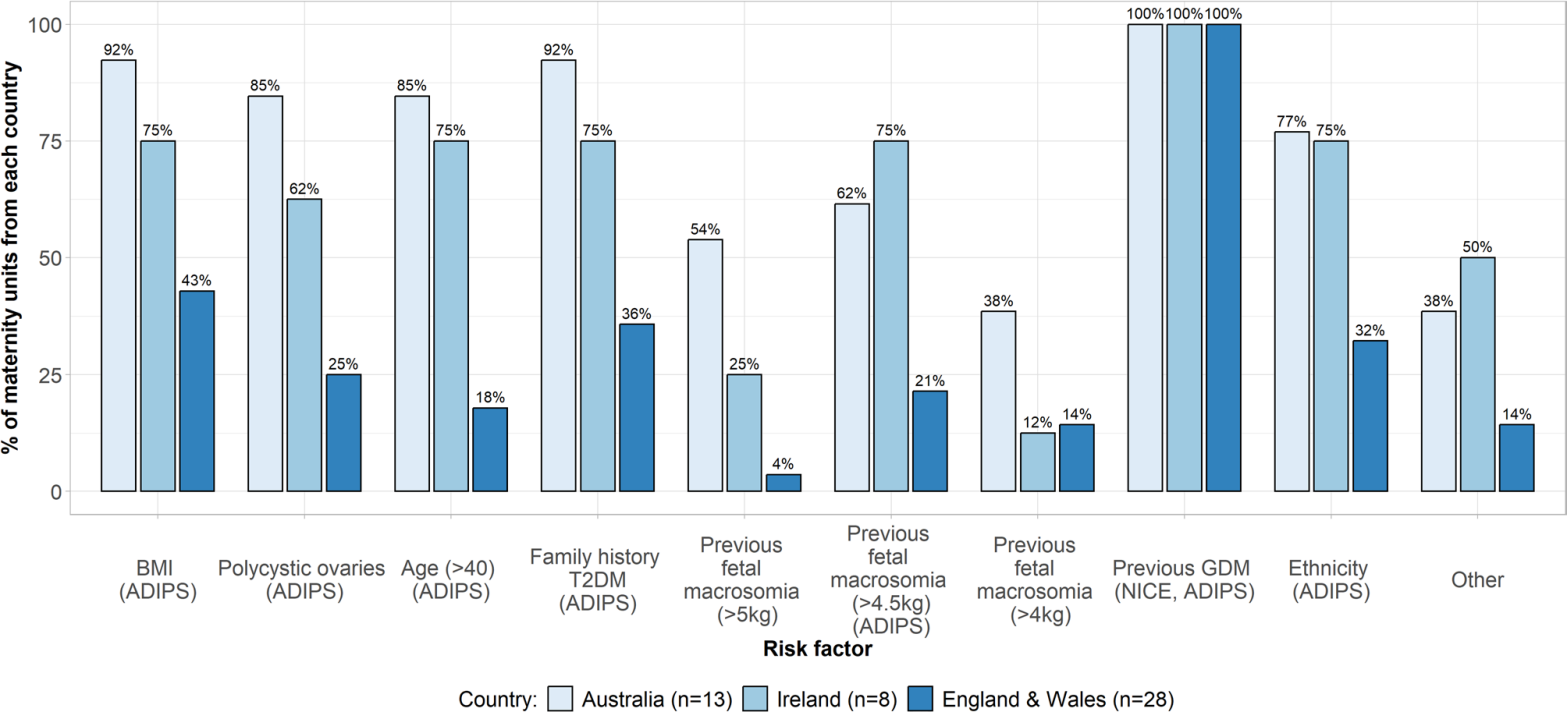
Risk factors used to select women for GDM testing in early pregnancy. ‘Other’ risk factors: England & Wales: polycystic ovarian syndrome, known pre-diabetes, glycosuria, in-vitro fertilization); Australia: thyroid disease or other chronic conditions, in-vitro fertilization previous stillbirth/unexplained perinatal death, pre-term birth, previous pre-eclampsia, age >35 years; Ireland: ‘symptoms’, glycosuria, previous perinatal death, long-term steroids, polyhydramnios in current pregnancy, current macrosomia, no response

*In early pregnancy* previous GDM was used to select women for GDM testing in all centres across countries, which aligns with RANZCOG/ADIPS and NICE guidelines, but differs from the Irish HSE guideline. Additional risk factors used varied between centres and countries. Figure 1 indicates risk factors used and whether they are included in the relevant national guideline.

NICE guidelines recommend early pregnancy testing only for women with previous GDM. Respondents in England and Wales indicated applying additional criteria including body mass index (BMI), family history of T2DM, and previous fetal macrosomia (with varying weight cut-offs). Australia showed between-centre variation for risk factors applied; in line with RANZCOG/ADIPS guidelines most used ethnicity, BMI, family history, age >40 years, polycystic ovary syndrome (PCOS), and previous fetal macrosomia (with varied cut-offs used, outside of guidance). Five Australian centres used ‘other’ criteria outside of guidelines including: thyroid disease or other chronic conditions, in-vitro fertilization, previous stillbirth/unexplained perinatal death, pre-term birth, previous pre-eclampsia, age >35 years (Fig. 1 caption). Most Irish centres described the use of risk factors stated in the HSE guidelines to select women for early testing (current macrosomia, polyhydramnios, glycosuria; see Fig. 1 caption). However, most Irish centres (5-6 of 8) also used risk factors outside of HSE guidelines including BMI, PCOS, age >40 years, family history of T2DM, previous fetal macrosomia (with varying weight cut-offs) and ethnicity. 

A reduced number of centres provided data about BMI thresholds used to select women for GDM testing (Australia = 12, England and Wales = 12, Ireland = 6; supplementary file 7). There was some variation within and between countries for the threshold used. The most commonly used in all three countries was BMI *≥* 30 kg/m^2^ (Australia *n* = 7/12, 58%; England and Wales *n* = 8/12, 75%; Ireland 4/6, 67%). A small number of centres in each country used other thresholds, including BMI > 25 kg/m^2^ (Australia *n* = 1/12, 17%; Ireland *n* = 1/6, 17%), BMI > 30 kg/m^2^ (Australia *n* = 2/12, 25%; England and Wales *n* = 1/12, 8%; Ireland *n* = 1/6, 17%), and one Irish centre (17%) reporting use of a threshold of 40 kg/m^2^.

**Fig. 2:**
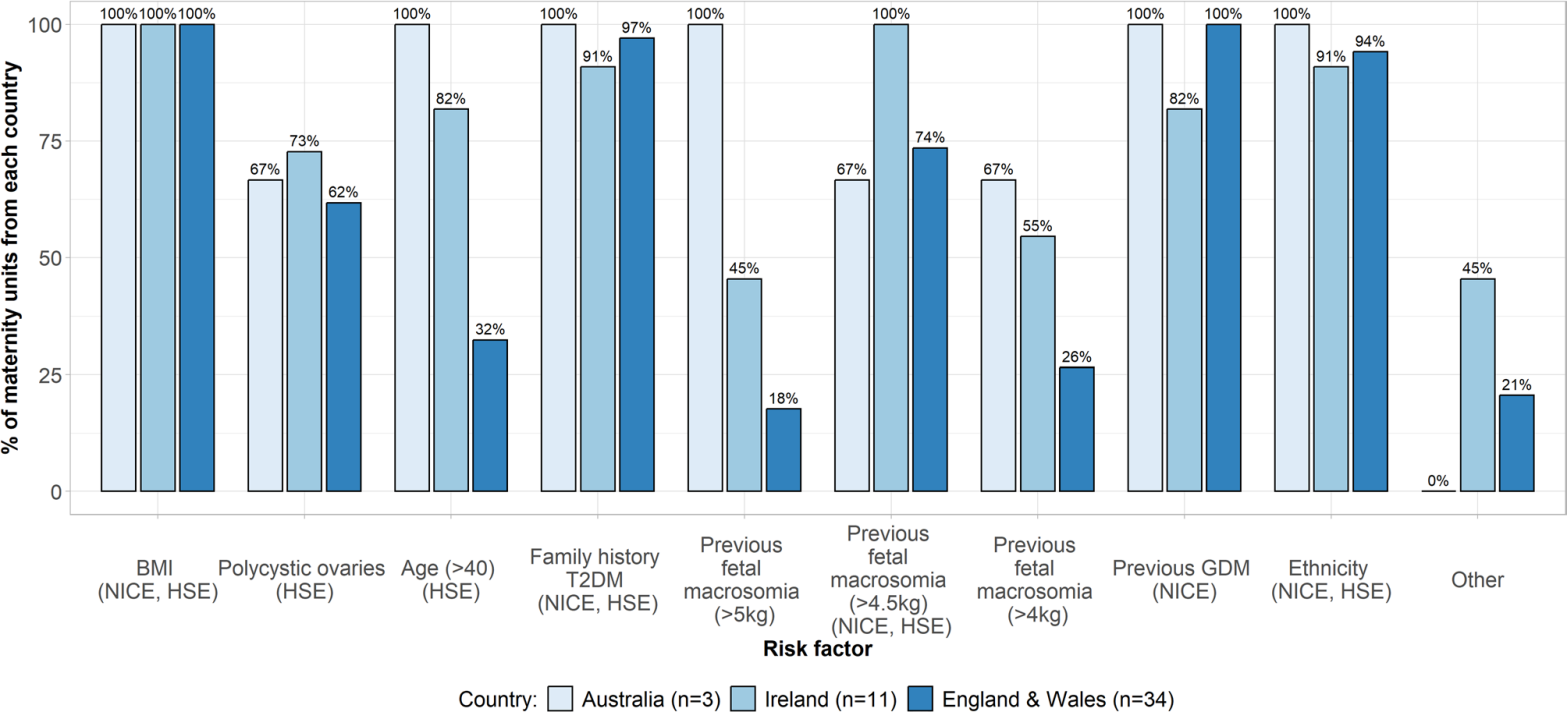
Risk factors used to select women for GDM testing at 24–28 weeks gestation. ‘Other’ risk factors: England & Wales: unexplained stillbirth, previous neonatal hypoglycemia, IVF, family history type 1 diabetes, antipsychotics, previous bariatric surgery; Ireland: previous unexplained perinatal death, current glycosuria, women on long term steroids, polyhydramnios and or macrosomia in existing pregnancy, previous stillbirth, previous IUD and steroid use, polyhydramnios at later scans, age > 35 years, and long-term corticosteroid use

*At 24–28 weeks gestation*, centres in England and Wales typically follow the NICE guideline, with most selecting women for GDM testing based on BMI, family history of diabetes, previous fetal macrosomia (with varied weight cut-offs, outside of guideline), ethnicity, and previous GDM (Fig. 2). Additional risk factors not included in the NICE guideline were PCOS and age > 40 years. A small number of centres report using ‘other’ criteria (see caption Fig. 2). Irish centres commonly used the HSE criteria, with over 70% (Ns *≥* 8/11) using BMI, PCOS, Age > 40, family history of T2DM, previous GDM, ethnicity, and macrosomia (with varied weight cut-offs- outside of guidelines). A small number of centres stated use of other HSE-guideline criteria including steroid use, current glycosuria, polyhydramnios, previous perinatal death (see Fig. 2 caption). Additionally, a small number use risk factors outside of HSE guidelines including age > 35 years and intrauterine device use. Three of the seven Australian centres using risk-factor based screening alongside universal screening reported the risk factors used: BMI, Age > 40, family history, macrosomia (with varying definitions), previous GDM and ethnicity. PCOS was used in two centres.

A small number of centres in Australia who reported selective testing (*n* = 3) and a larger number of centres in England and Wales (*n* = 32) and Ireland (*n* = 10), where selective testing is recommended in guidelines, reported the BMI cut-offs used to select women for GDM testing in later pregnancy (see supplementary file 7). In line with their respective national guidelines, most centres across all three countries used a threshold of BMI *≥* 30 kg/m^2^. In Ireland and England and Wales a smaller number of centres also used BMI > 35 kg/m^2^ (England and Wales *n* = 5/32, 16%; Ireland *n* = 3/10, 30%), and in Ireland and Australia, one centre used a threshold of BMI > 25 kg/m^2^ respectively (Ireland *n* = 1/10, 10%; Australia *n* = 1/3, 33%).

### GDM tests used

#### Early pregnancy testing

In England and Wales NICE guidelines recommend a GTT or self-monitoring of blood glucose for women with previous GDM. RANZCOG/ADIPS guidelines recommend a GTT or HbA1c. In Ireland HSE guidelines recommend they are treated as though diagnosed with GDM (see S1).

**Fig. 3:**
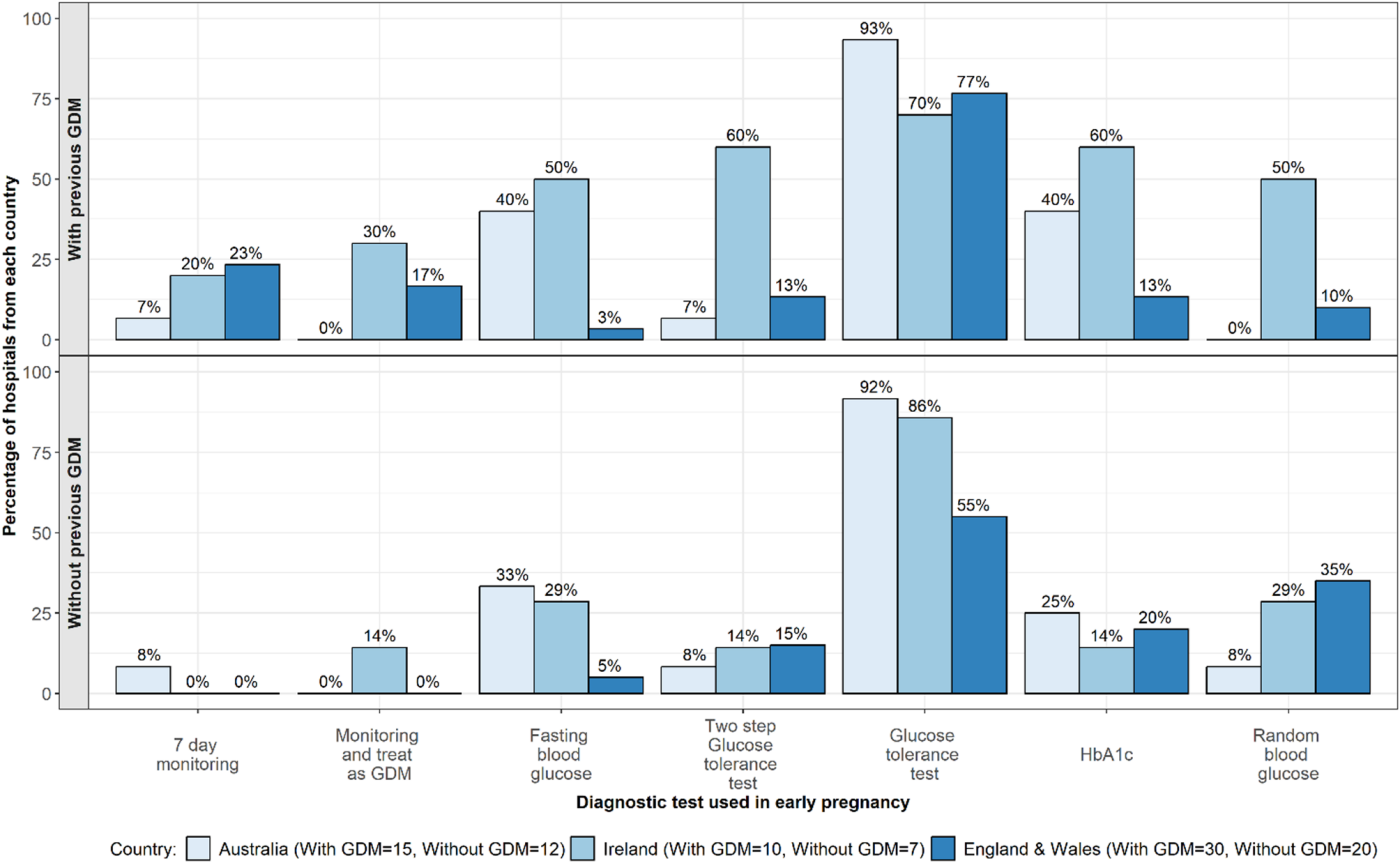
Blood glucose tests used to diagnose GDM in early pregnancy

In women with previous GDM Over 70% of centres across all three countries used a GTT to diagnose GDM in women with previous GDM in early pregnancy (Fig. 3). There was greater diagnostic test variation seen in Ireland compared with Australia and England and Wales. Over 50% (n *≥* 5/10) of Irish centres used the 2-step GTT, HbA1c, random blood glucose (RBG) or fasting blood glucose (FBG). In England and Wales, 23% (*n* = 7/30) and 17% (*n* = 5/30) of centres used 7-day monitoring or monitoring and treating women as though they have GDM respectively, with a small number of centres using tests outside of NICE guidelines. A FBG and HbA1c was used in 40% (*n* = 6/15) of Australian centres respectively, with very limited use of other tests.

*In women without previous GDM*: A GTT was the most commonly used test as per national guidelines in all three countries (see Fig. 3). In England and Wales 15%−35% (*n* = 3–7/20) used a 2-step GTT, HbA1c or RBG, which are not recommended. In Australia, 25%−33% (*n* = 3–4/12) used FBG and HbA1c as stated in RANZCOG/ADIPS guidelines. In Ireland, 29% (*n* = 2/7) of centres indicated using a FBG or RBG, with a small number of centres using monitoring and treating as GDM, two-step GTT and HbA1c, outside of HSE guidance.

#### At 24–28 weeks gestation

**Fig. 4:**
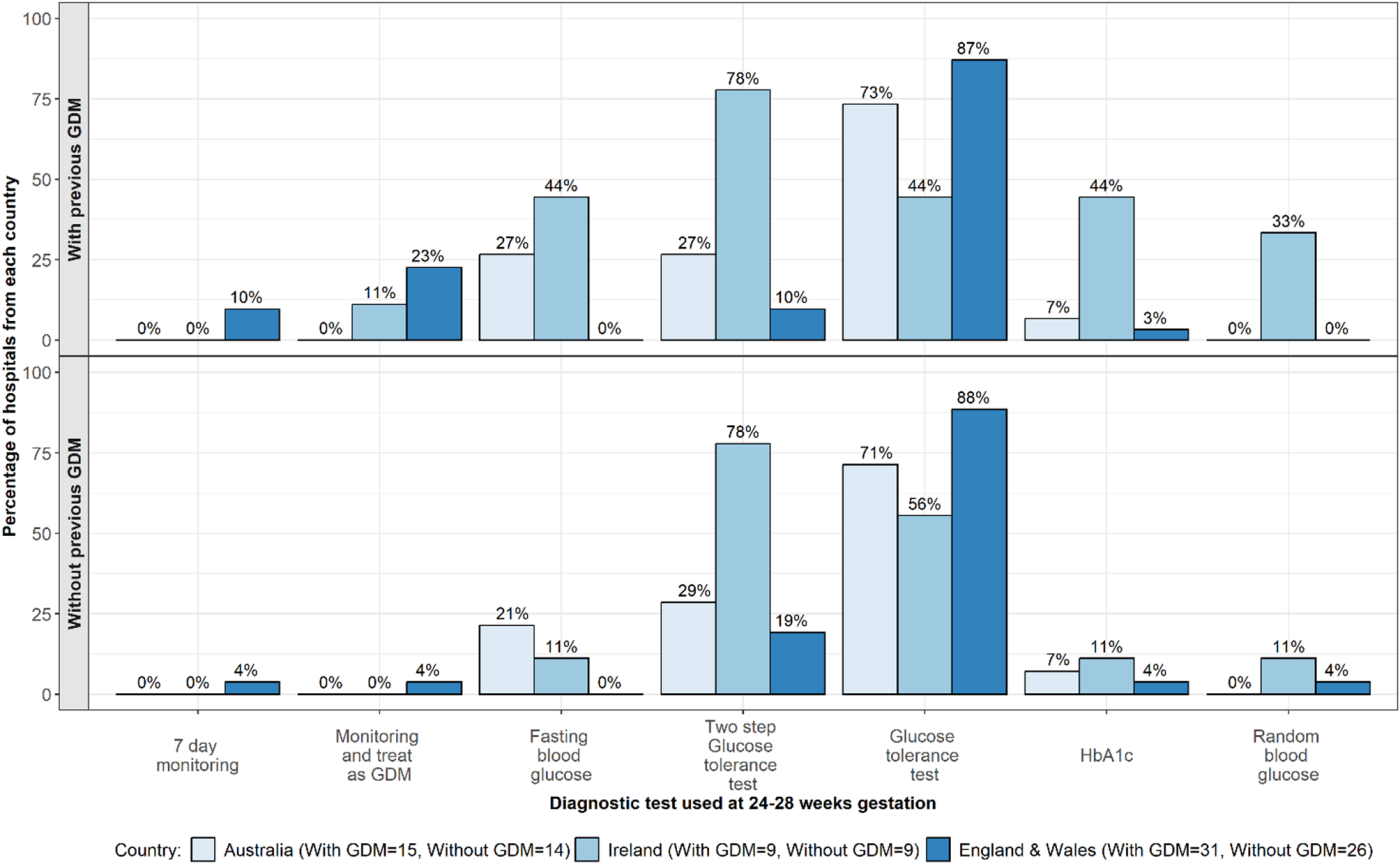
Blood glucose tests used to diagnose GDM at 24–28 weeks gestation

*In women with previous GDM* the GTT was the most commonly used test in England and Wales and Australia (per NICE and RANZCOG/ADIPS guidelines; See Fig. 4). In England and Wales, 23% (*n* = 7/31)of centres reported monitoring and treating as GDM for women with previous GDM (per NICE guidelines). In Ireland the 2-step GTT was the most commonly used test (*n* = 7/9, 78%; outside of HSE guidelines), and there was more variation in tests used, with 44% (*n* = 4/9) of centres using the single step GTT (as per HSE guidelines) and HbA1c, alongside FBG and RBG.

*In women without previous GDM*, the GTT was the most commonly used test in Australia (*n* = 12/14, 71%) and England and Wales (*n* = 23/26, 88%). A wider range of tests, outside of national guidelines, was used in Ireland. The 2-step GTT was the most commonly used test (as per HSE guidelines; *n* = 7/9, 78%), alongside the single step GTT, with a small number using RBG, FBG and HbA1c. A small number of centres in England and Walesused the 2-step GTT, HbA1c and RBG (outside of NICE guidelines). In Australia, additional tests used in a small number of centres included the 2-step GTT, HbA1c and FBG (outside of RANZCOG/ADIPS guidelines).

### Diagnostic criteria

Most Australian centres (*n* = 14/15, 93%) used the ADIPS or IADPSG-WHO criteria (which are the same) to diagnose GDM (see S1 for thresholds). The NICE guideline was used in most centres in England and Wales (*n* = 24/31, 77%), but other guidelines used included an adjusted version of the NICE guideline and the IADPSG/WHO (*n* = 3/31, 10% respectively; see supplementary file 8).

In Ireland, there was more variation in diagnostic guidelines used; 8/10 centres (80%) used NICE and 3/10 (30%) used the IADPSG-WHO diagnostic thresholds (which align with HSE guidelines). ‘Other’ guidelines used across all three countries were typically local guidelines or modifications to existing national guidelines (see caption supplementary file 8).

### GDM post-diagnosis care

A smaller number of centres completed data about care pathways. Medication, dietary, physical activity, blood glucose monitoring education and additional growth scanning were offered across almost all centres (92–100%; see supplementary file 9).

Where lifestyle modification failed, biguanides (Metformin) were the commonest first-line medication in England and Wales (as per NICE guidelines) and Ireland (where the HSE recommends insulin as the first line treatment; Table 1). Insulin was commonest in Australia, with biguanides the preferred second-line treatment. Sulfonylureas were infrequently used in all three countries.

**Table 1:** First and second-line medications for GDM management following failure of lifestyle modification by country

	Australia (*n* = 10)		England & Wales (*n* = 21)	Ireland (*n* = 7)
**First line medications**				
Biguanides	3 (30.0%)		18 (85.7%)	5 (71.4%)
Insulin	7 (70.0%)		2 (9.5%)	3 (42.9%)
Sulfonylureas	0 (0.0%)		1 (4.8%)	0 (0.0%)
**Second line medications**				
Biguanides	5 (50.0%)		2 (9.5%)	1 (14.3%)
Insulin	2 (20.0%)		19 (90.5%)	5 (71.4%)
Sulfonylureas	2 (20.0%)		1 (4.8%)	1 (14.3%)

#### Timing of birth

Table 2 illustrates gestation at which birth via induction of labour (IoL) or caesarean section (CS) typically planned according to management strategy (lifestyle and medication type). For lifestyle-managed GDM, birth typically planned at 39–40 weeks in Australia and Ireland (per HSE guidelines) and at 40–41 weeks in England and Wales (per NICE guidelines). Birth typically plannedat 38–39 weeks gestation for insulin-managed GDM. Most centres indicated that birth typically planned at 39–40 weeks for metformin-managed GDM in England and Wales and Ireland, and 38–39 weeks gestation in Australia.

**Table 2:** Gestation at which birth typically occurs for women with GDM across countries, according to treatment

Gestation	Australia (*n* = 10)	England + Wales (*n* = 22)	Ireland (*n* = 7)
**Lifestyle**			
37–38 weeks	0 (0.0%)	2 (9.1%)	3 (42.9%)
38–39 weeks	2 (20.0%)	5 (22.7%)	4 (57.1%)
39–40 weeks	6 (60.0%)	10 (45.5%)	6 (85.7%)
40–41 weeks	4 (40.0%)	16 (72.7%)	4 (57.1%)
**Metformin**			
37–38 weeks	0 (0.0%)	4 (18.2%)	3 (42.9%)
38–39 weeks	6 (60.0%)	10 (45.5%)	4 (57.1%)
39–40 weeks	5 (50.0%)	14 (63.6%)	5 (71.4%)
40–41 weeks	1 (10.0%)	5 (22.7%)	1 (14.3%)
**Insulin**			
37–38 weeks	4 (40.0%)	8 (36.4%)	3 (42.9%)
38–39 weeks	8 (80.0%)	13 (59.1%)	5 (71.4%)
39–40 weeks	5 (50.0%)	8 (36.4%)	4 (57.1%)
40–41 weeks	0 (0.0%)	2 (9.1%)	1 (14.3%)

## Discussion

This survey of GDM-related care practices across three countries with similar public healthcare systems identified considerable between-country variations in guidelines used, testing indications, diagnostic tests and criteria used, and post-diagnosis care pathways. Importantly, we also identified considerable practice variation within countries and that care frequently deviates from national guideline recommendations.

While most maternity centres reported using their national guideline to determine who is offered GDM testing, several centres reported using other guidelines, particularly in Ireland. We also noted that many centres across countries used risk factors for selective GDM testing beyond those in their national guideline. For example, several centres in England and Wales employed risk factors outside of NICE guidelines [14] for GDM testing in early and later pregnancy (e.g. PCOS, maternal age, pre-diabetes), although it should be noted that some of these risk factors appear in the other countries’ guidelines [12, 13]. Similarly, universal testing was reported in a small number of centres in Ireland and England and Wales, where it is not recommended in guidelines. This variation in screening criteria, both between and within countries, may lead to inequitable access to GDM screening and potentially missed diagnoses according to maternity centre attended.

Similar variations in diagnostic tests used and adherence to national guidelines related to them was also observed, particularly for women with previous GDM and in the use of one or two-step GTTs. In all three countries a GTT is recommended in national guidelines, with greater or lesser provision for use of other tests, which provides an explanation for some of the variation in tests used. The evidence for the accuracy of various diagnostic tests is conflicting. For example, HbA1c has been found to be more divergent from the GTT, i.e. potentially less accurate, in later pregnancy compared with early pregnancy [22]. However, a recent prospective study in India and Kenya demonstrated that an early pregnancy HbA1c was independently associated with incidence of GDM at 24–28 weeks gestation, potentially reducing the need for tests in later pregnancy such as GTTs [23]. A systematic review concluded that random plasma glucose was insufficiently sensitive and specific as a GDM screening test [24]. This variation in diagnostic tests used likely reflects the lack of a ‘gold standard’ GDM test and may also reflect patient preferences, as some report poor experiences with GTT [25]. While the GTT has limitations [26], using tests with compromised sensitivity and outside of current guidelines may result in missed opportunities to diagnose and treat GDM with potential adverse impact on perinatal outcomes. There is increasing interest in use of continuous glucose monitoring devices to diagnose GDM [27, 28]; further evidence is required to explore their sensitivity and specificity for diagnosis. We did not explore why women who do not meet the national guideline criteria are being tested, nor why non-recommended tests are being used. Variations in implementation of national guidelines are likely to be multifactorial, involving individual healthcare practitioners’ knowledge and agreement with them, and organisational factors [16]. To address barriers to using guidelines, and increase their use, future work should explore the reasons for non-adherence to national GDM guidelines for diagnosis.

We observed variations in care offered between and within countries, with some non-adherence to national guidelines, particularly in first-line medication use and timing of birth. The variation in first-line medication use is well-documented [29] with insulin typically used where lifestyle modification is does not achieve glucose targets. This is still the case in countries such as the USA [30]. Randomised controlled trials (RCTs) have demonstrated metformin’s efficacy in managing GDM compared with insulin [29, 31], and its use is supported in some national GDM treatment guidelines [14]. However, organisations such as the American College of Obstetricians and Gynecologists, HSE and IADPSG do not endorse metformin as a first-line therapy due to potential adverse offspring outcomes from in-utero exposure, including lower birth weight, accelerated infant growth and higher mid-childhood BMI [32]. The ongoing Metformin in Pregnancy Study aims to use individual patient data meta-analysis to clarify the efficacy and safety of metformin use in pregnancy and to identify relevant knowledge gaps, which can inform treatment guidelines [33]. 

Optimal birth timing for women with GDM remains controversial. Our findings align with previous studies showing wide variation within and between countries for timing of birth [34]. A single RCT of insulin-treated women found fewer large for gestational age (LGA) cases with IoL at 38 weeks compared to expectant management, with no increased risk of caesarean delivery, shoulder dystocia or neonatal complications [35]. However, these results may not be generalisable to all women with GDM given the inclusion of women with pre-gestational diabetes and only those treated with insulin.

### Implications for clinical practice, research and policy

The variations in GDM care practices identified in this study have significant implications. The differences seen in diagnostic criteria, tests and care pathways between countries are likely to result in considerable differences in diagnosis, prevalence, treatment and likely outcomes. Economic factors such as the cost of universal screening and treatment are potential barriers to a standardised approach to GDM diagnosis, treatment and pregnancy management. However, the practice variations within countries is of particular significance. This creates a potential ‘lottery’ effect, resulting in risk of under- or over-diagnosis of GDM depending on maternity centre attended. Undiagnosed GDM is associated with a two- to four-fold risk of macrosomia and maternal and infant morbidity [36]. Conversely, universal GDM testing is linked with increased rates of diagnosis [37, 38], potentially leading to higher health system costs, burden and higher rates of interventions. Additionally, variations in post-diagnosis medication use and timing of delivery can impact clinical outcomes for the woman and child. It is vital that future research explores the reasons for, and comparative clinical and cost implications of these variations across populations to better inform GDM care. Additionally, the potential for personalised risk prediction approaches to more accurately identify those at risk of GDM and those who are most at risk of adverse pregnancy outcomes requires investigation [39]. The goal should be to develop evidence-based care practices that are adopted into care pathways to ensure standardisation of care across centres and effective GDM management, while optimising resource use.

### Strengths and limitations

We recruited multidisciplinary healthcare professionals across geographic locations in three countries, giving a perspective of differing practices within and between the participating countries. Limitations include the response rate, with variation in the proportion of centres responding across countries, and attrition, resulting in reduced data about post-diagnosis care practices. While a high response rate in Ireland supports the validity of findings, the lower response rate in Australia and England and Wales could reduce generalisability of the findings to the broader healthcare system in each country, and could be impacted by response bias [40], wherein there are systematic differences in the practices of those centres that responded and those that did not.

Some centres provided multiple responses that contradicted one another. This may be due to receiving responses from more than one healthcare professional group, who work in different parts of the care pathway and may have greater or lesser knowledge about differing aspects of care provided. Screening and diagnosis, for example, may be outside the scope of practice for that group and therefore the responses of individuals not directly involved in that aspect of care may not accurately reflect practice. We aimed to mitigate this by enabling respondents to not respond to questions where they lacked knowledge relevant to the answer. These discrepancies could also indicate individuals’ differing practices within centres and it is also possible that their responses reflect planned practice rather than what happens in reality. We did not have a sufficient sample size to conduct meaningful detailed analyses to explore whether there were differences in responses according to job role within centres.

## Conclusion

Effective diagnosis and treatment of GDM is crucial for improving short- and long-term perinatal and maternal health outcomes, including reduced morbidity, mortality, and healthcare costs. We identified variations in GDM screening, diagnosis and care pathways within and between countries with universal public healthcare systems and established healthcare guidelines, and non-adherence to these national guidelines in responding centres. These variations within countries have important implications for equitable and effective GDM management, potentially impacting short- and long-term perinatal and maternal health outcomes. Future research should identify optimal evidence-based care practices and develop effective guideline implementation strategies to increase standardisation of care within countries. Ultimately, these efforts aim to ensure equitable, effective and cost-efficient GDM care, with potential benefits for reduced maternal and offspring morbidity and mortality.

## Supplementary Information

Supplementary Material 1. Supplementary file 1: Testing and treatment guidelines in the UK, Australia and Ireland

Supplementary Material 2. Supplementary file 2: Checklist for Reporting Results of Internet E-Surveys (CHERRIES)

Supplementary Material 3. Supplementary file 3: Participant job roles

Supplementary Material 4. Completion rate for questions

Supplementary Material 5. Supplementary file 5: Guidelines used who is tested for GDM

Supplementary Material 6.Supplementary file 6: Universal versus selective screening

Supplementary Material 7. Supplementary file 7: BMI thresholds for GDM screening

Supplementary Material 8. Supplementary file 8: Diagnostic guidelines used

Supplementary Material 9. Supplementary file 9: Care offered to women diagnosed with GDM.

## Data Availability

The data that support the findings of this study are available from the authors, but restrictions may apply. Data may therefore be available upon reasonable request to the corresponding author, with appropriate ethical permissions in place.

## Abbreviations

ADIPS: Australasian Diabetes in Pregnancy Society
BMI: Body mass index
CS: Caesarean section
FBG: Fasting blood glucose
GDM: Gestational Diabetes Mellitus
GTT: Glucose tolerance test
HbA1c: Glycosylated haemoglobin
HSE: Health and Safety Executive (Ireland)
IADPSG: International Association of Diabetes and Pregnancy Study Groups
IDB2B&Me: Impact Diabetes Bump2Baby&Me
IoL: Induction of labour
LGA: Large for gestational age
NICE: National Institute for Health and Care Excellence (UK)
PCOS: Polycystic ovarian syndrome
RCT: Randomised controlled trial
RBG/RPG: Random blood/plasma glucose
RANZCOG: Royal Australian and New Zealand College of Obstetrics and Gynaecology
T2DM: Type 2 diabetes mellitus
UK: United Kingdom of Great Britain and Northern Ireland
WHO: World Health Organization
