# Supplementary material for: Variation in gestational diabetes diagnosis and care practices in maternity services in three high-income countries; a cross-sectional survey
Source: BMC Pregnancy Childbirth. 2025 Dec 6;26:165. doi: 10.1186/s12884-025-08472-5 (PMC12908269; doi:10.1186/s12884-025-08472-5)
Supplement: Supplementary file 1 — Supplementary Material 1. Supplementary file 1: Testing and treatment guidelines in the UK, Australia and Ireland [file 12884_2025_8472_MOESM1_ESM.docx]

| **UK- NICE**  [**Recommendations \| Diabetes in pregnancy: management from preconception to the postnatal period \| Guidance \| NICE**](https://www.nice.org.uk/guidance/ng3/chapter/Recommendations#gestational-diabetes) | **Ireland- HSE**  [**Guidelines for the Management of Pre-gestational and Gestational Diabetes Mellitus from Pre-conception to the Postnatal period 2010**](https://www.hse.ie/eng/services/list/2/primarycare/east-coast-diabetes-service/management-of-type-2-diabetes/diabetes-and-pregnancy/guidelines-for-the-management-of-pre-gestational-and-gestational-diabetes-mellitus-from-pre-conception-to-the-postnatal-period.pdf) | **Australia- RANZCOG/ADIPS**  [**ADIPS Consensus Guidelines for the Testing and Diagnosis of Hyperglycaemia in Pregnancy in Australia and New Zealand (modified November 2014)**](https://www.adips.org/downloads/2014ADIPSGDMGuidelinesV18.11.2014_000.pdf)  [**RANZCOG Diagnosis of Gestational Diabetes Mellitus**](https://ranzcog.edu.au/wp-content/uploads/Diagnosis-Gestational-Diabetes-Mellitus.pdf) |
| --- | --- | --- |
| **Who is offered GDM testing** | | |
| **Selected women with risk factors:**   - previous gestational diabetes - BMI above 30 kg/m^2^ - previous macrosomic baby weighing 4.5 kg or more - family history of diabetes (first degree relative with diabetes) - an ethnicity with a high prevalence of diabetes (ethnicities not stated).   Consider testing to exclude gestational diabetes in women who have the following reagent strip test results during routine antenatal care:   - glycosuria of 2+ or above on 1 occasion - glycosuria of 1+ or above on 2 or more occasions. | **Selected women with risk factors:**   - Family history of diabetes in a first degree relative - Body mass index ≥30kg/m2 - Maternal age ≥ 40years - Previous unexplained perinatal death - Current glycosuria - Women on long term steroids - Previous delivery of a baby weighing ≥4.5kg - Polycystic Ovary Syndrome - Polyhydramnios and/or macrosomia in existing pregnancy - Ethnicity associated with a high prevalence of diabetes: (India/ Pakistan/ Bangladesh/ Black Caribbean/ Saudi Arabia/ United Arab Emirates/ Iraq/ Jordan/ Syria/ Oman/ Qatar/ Kuwait/ Lebanon/Egypt)   While some centres re-screen women with a history of gestational diabetes with a 75g OGTT at 24-28 weeks gestation, it is recommended that the woman be referred at booking for combined diabetes/obstetric antenatal care. | **Universal screening:** All women not previously known to have pre pregnancy diabetes or hyperglycemia in pregnancy (GDM) should be tested at 24-28 weeks gestation  **Women not known to have pre-existing glucose abnormalities, but with risk factors for hyperglycaemia in pregnancy** **should be tested early in pregnancy**:   - Previous hyperglycaemia in pregnancy - Previously elevated blood glucose level - Maternal age ≥40 years - Ethnicity: Asian, Indian subcontinent, Aboriginal, Torres Strait Islander, Pacific Islander, Maori, Middle Eastern, non-white African - Family history DM (1st degree relative with diabetes or a sister with hyperglycaemia in pregnancy) - Pre-pregnancy BMI > 30 kg/m2 - Previous macrosomia (baby with birth weight > 4500 g or > 90th centile) - Polycystic ovarian syndrome - Medications: corticosteroids, antipsychotics |
| **Recommended test** |  |  |
| 75g 2-hour oral glucose tolerance test (OGTT) to test for gestational diabetes in women with above risk factors.  **In women who have had GDM in a previous pregnancy offer:**   - early self monitoring of blood glucose **or** - a 75-g 2hour OGTT as soon as possible after booking (whether in the first or second trimester), and a further 75-g 2hour OGTT at 24 to 28 weeks if the results of the first OGTT are normal.   **In women with the other risk factors, offer a 75g OGTT at 24-28 weeks**  Do not use fasting plasma glucose, random blood glucose, HbA1c, glucose challenge test or urinalysis for glucose to assess the risk of developing gestational diabetes. | 75g 2-hour OGTT at 24-28 weeks’ gestational age for women with above risk factors.  Test if GDM is suspected at an earlier or later gestation than 24-28 weeks, on the basis of fetal macrosomia, polyhydramnios or glycosuria. If negative at an early gestation, repeat the test between 24-28 weeks gestation | **All women not previously known to have pre-pregnancy diabetes:** 75g OGTT at 24-28 weeks gestation  **Women not known to have pre-existing glucose abnormalities but with risk factors should be tested early in pregnancy:** The method of testing must be based on clinical judgement, local health care policy and possible risk stratification (see section requiring further research). Women deemed at higher risk should ideally have a pregnancy OGTT or a HbA1c. |
| **Diagnostic criteria** |  |  |
| Diagnose GDM if the woman has either:   - a fasting plasma glucose (FPG) of 5.6 mmol/L or above **or** - a 2-hour FPG level of 7.8 mmol/L or above | Diagnose GDM if one or more values are met or exceeded:   - FPG of 5.1mmol/L - 1-hour FPG of 10.0mmol/L - 2-hour FPG of 8.5mmol/L | Diagnosis of GDM should be based on any one of:   - FPG of 5.1–6.9 mmol/L - 1-hour post 75 g oral glucose load of ³10.0 mmol/L - 2-hour post 75 g oral glucose load 8.5–11.0 mmol/L |
| **Medication** |  |  |
| - If blood glucose targets are not met with diet and exercise changes within 1 to 2 weeks, offer metformin. - If metformin is contraindicated or unacceptable to the woman, offer insulin. - If blood glucose targets are not met with diet and exercise changes plus metformin, offer insulin as well. | - If treatment targets cannot be met with diet and physical activity alone, insulin therapy must be considered - Insulin therapy must be considered when the glycaemic target values are exceeded on two or more occasions within a 1-2 week interval, especially in the presence of confirmed macrosomia | - Insulin therapy offered as first line medication. |
| **Birth** |  |  |
| - Advise women with gestational diabetes to give birth no later than 40 weeks plus 6 days. - Offer elective birth by induced labour or (if indicated) by caesarean section to women who have not given birth by this time. - Consider elective birth before 40 weeks plus 6 days for women with gestational diabetes who have maternal or fetal complications. | - In the setting of excellent glycaemic control, adherence to treatment and absence of maternal and fetal compromise, women with diabetes may await spontaneous labour up to 39-40 weeks gestation. - The frequency of fetal monitoring should be increased if the pregnancy is allowed to progress beyond 40 weeks’ gestation. |  |
