# Supplementary material for: Variation in gestational diabetes diagnosis and care practices in maternity services in three high-income countries; a cross-sectional survey
Source: BMC Pregnancy Childbirth. 2025 Dec 6;26:165. doi: 10.1186/s12884-025-08472-5 (PMC12908269; doi:10.1186/s12884-025-08472-5)
Supplement: Supplementary file 3 — Supplementary Material 3. Supplementary file 3: Participant job roles [file 12884_2025_8472_MOESM3_ESM.docx]

**Supplementary file 3:** Participant job role by country

| Job role | Australia, N = 19 | England and Wales, N = 45 | Ireland, N = 38 |
| --- | --- | --- | --- |
|  |  |  |  |
| Diabetes Specialist Nurse | 7 (36.8%) | 0 (0%) | 0 (0%) |
| Dietitian | 4 (21.1%) | 2 (4.4%) | 9 (23.7%) |
| Endocrinologist | 1 (5.3%) | 9 (20.0%) | 0 (0%) |
| Midwife | 0 (0%) | 11 (24.4%) | 20 (52.6%) |
| Obstetrician | 5 (26.3%) | 22 (48.9%) | 9 (23.7%) |
| Other* | 2 (10.5%) | 1 (2.2%) | 0 (0.0%) |

*Other: Researcher, Lecturer, Diabetes Educator
