# Supplementary material for: Variation in gestational diabetes diagnosis and care practices in maternity services in three high-income countries; a cross-sectional survey
Source: BMC Pregnancy Childbirth. 2025 Dec 6;26:165. doi: 10.1186/s12884-025-08472-5 (PMC12908269; doi:10.1186/s12884-025-08472-5)
Supplement: Supplementary file 4 — Supplementary Material 4. Completion rate for questions [file 12884_2025_8472_MOESM4_ESM.docx]

Supplementary file 4: Completion rate for questions

| Question of interest | Number of responses | Completion rate |
| --- | --- | --- |
| Key questions |  |  |
| Guidelines used for screening | 102 | 100% |
| Timing of screening | 101 | 99% |
| Criteria used to select women for GDM screening in first trimester | 64 | 63% |
| Criteria used to select women for GDM screening at 24-28 weeks gestation | 78 | 76% |
| Glucose test used to diagnose GDM in early pregnancy | 84 | 82% |
| Glucose test used to diagnose GDM at 24-28 weeks gestation | 84 | 82% |
| Guidelines to diagnose GDM | 81 | 79% |
