# Supplementary figures and images for: Variation in gestational diabetes diagnosis and care practices in maternity services in three high-income countries; a cross-sectional survey
Source: BMC Pregnancy Childbirth. 2025 Dec 6;26:165. doi: 10.1186/s12884-025-08472-5 (PMC12908269; doi:10.1186/s12884-025-08472-5)

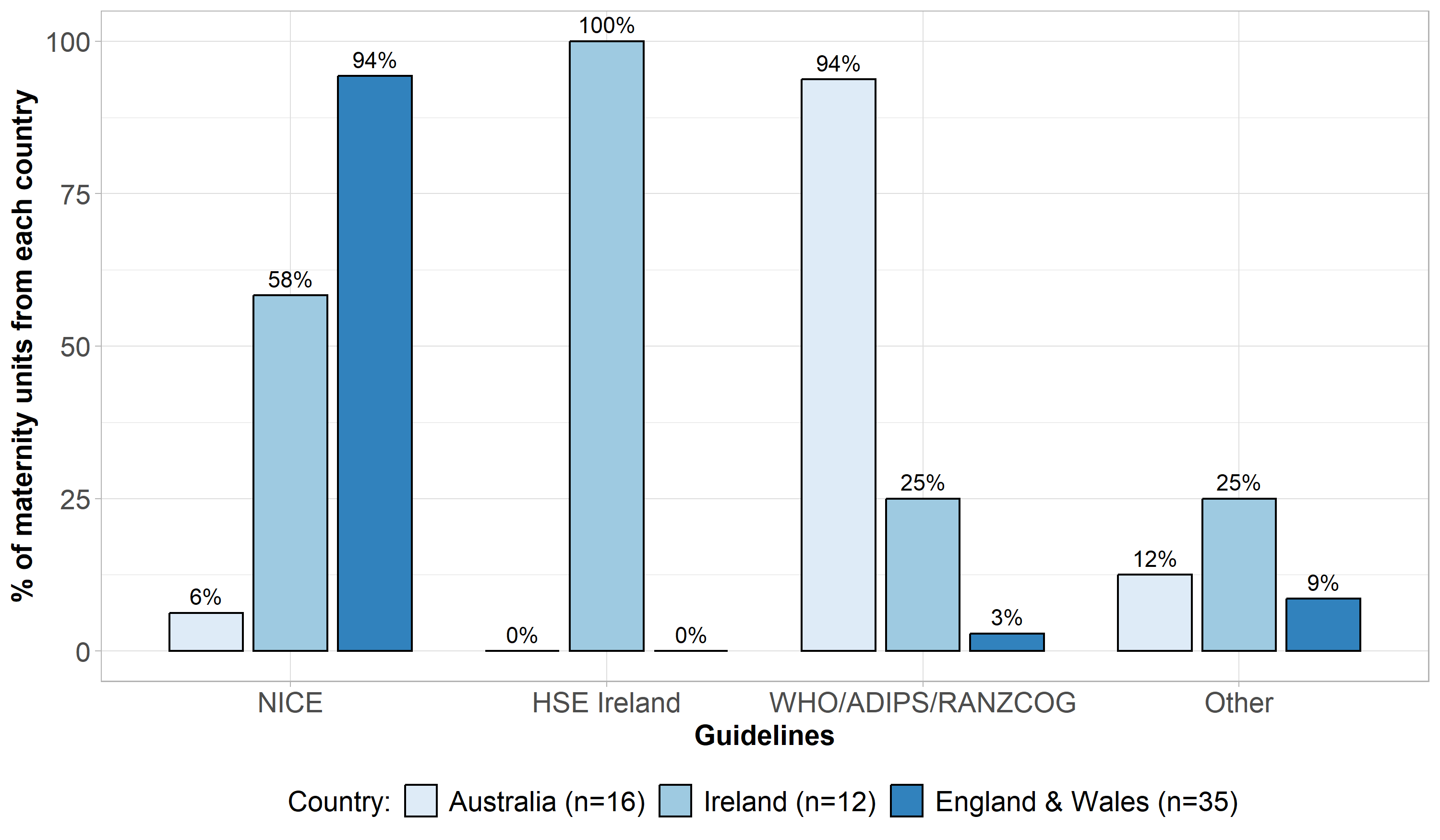

Supplement: Supplementary file 5 — Supplementary Material 5. Supplementary file 5: Guidelines used who is tested for GDM [file 12884_2025_8472_MOESM5_ESM.png]

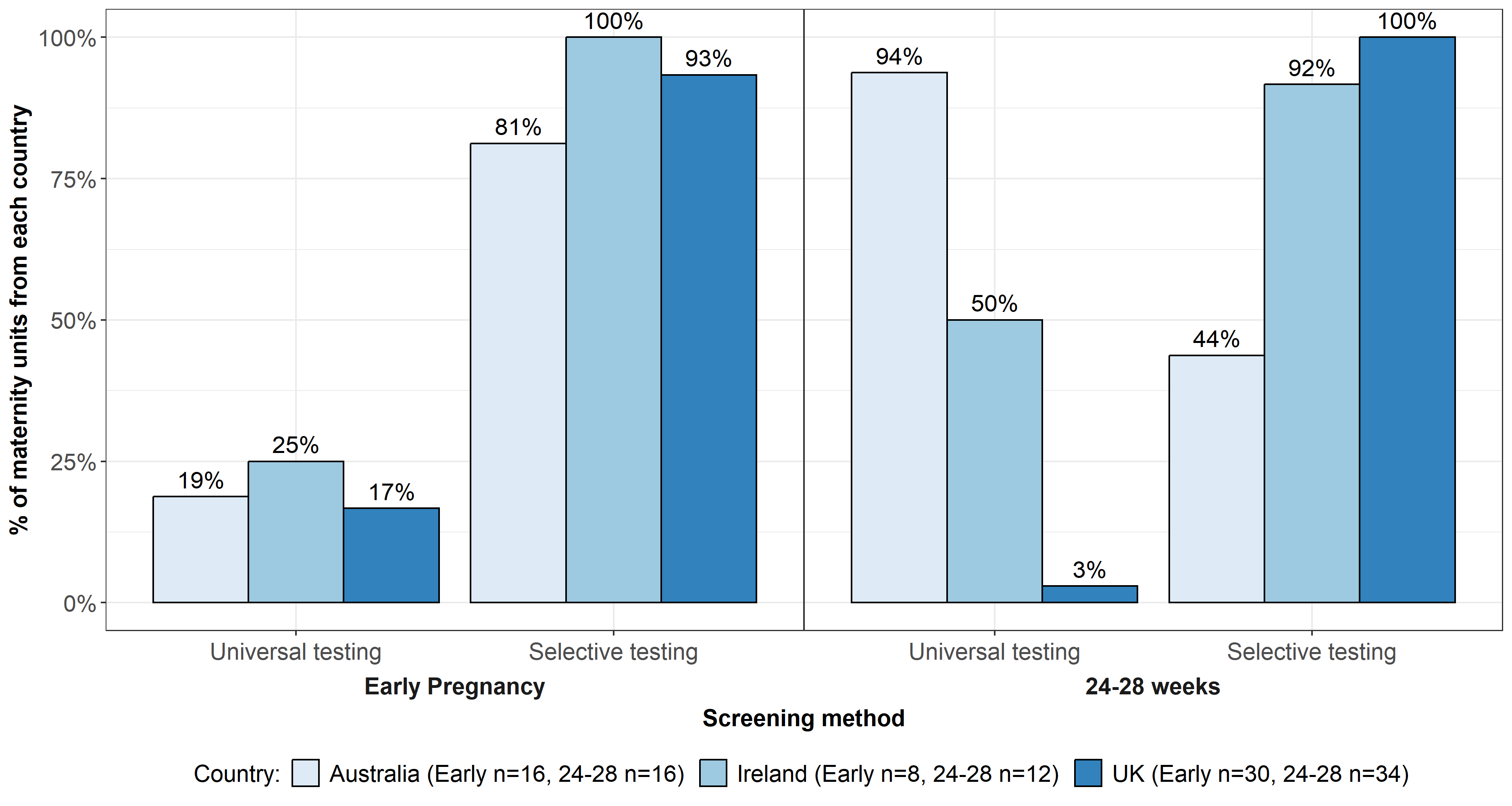

Supplement: Supplementary file 6 — Supplementary Material 6.Supplementary file 6: Universal versus selective screening [file 12884_2025_8472_MOESM6_ESM.png]

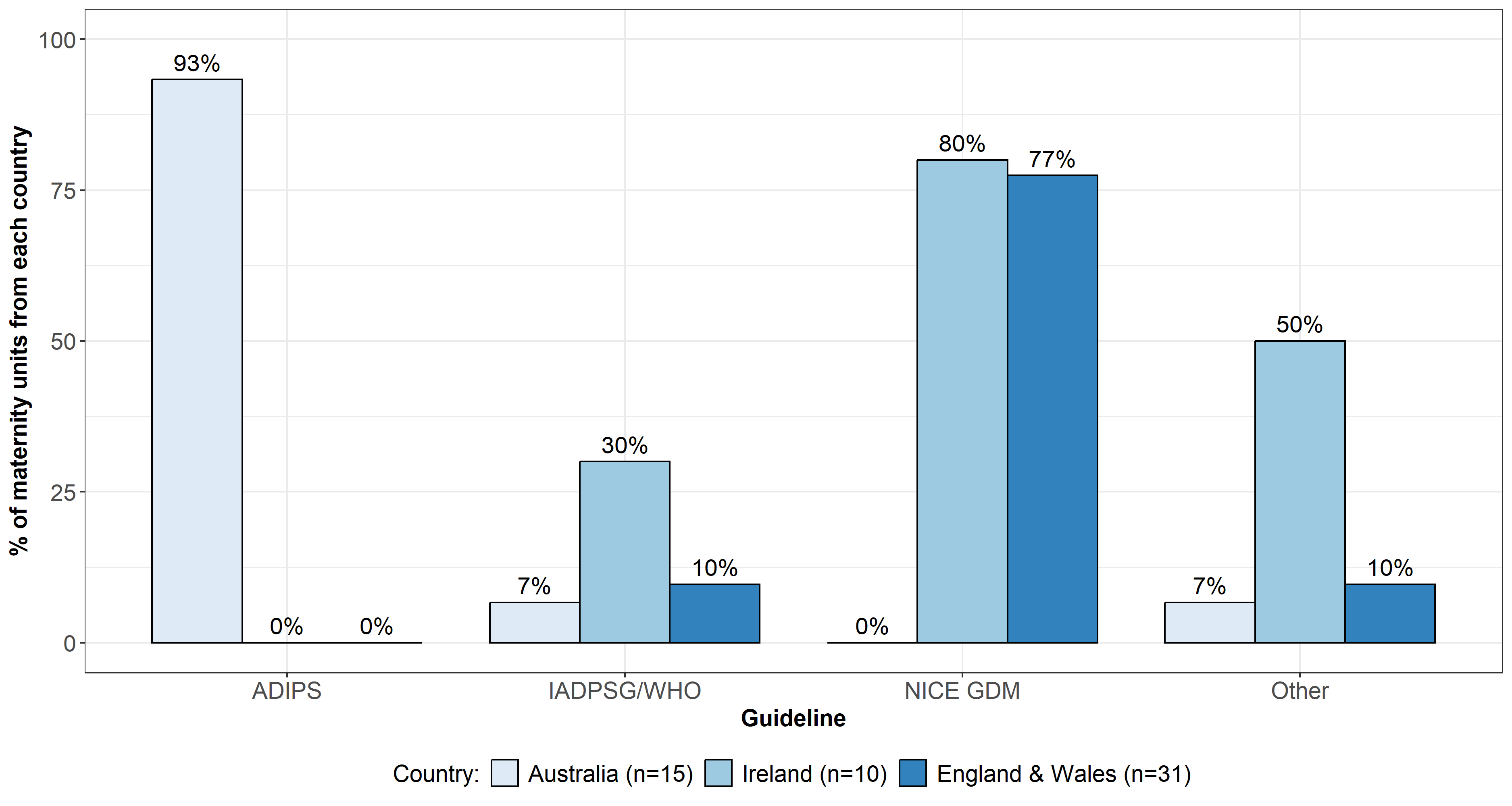

Supplement: Supplementary file 8 — Supplementary Material 8. Supplementary file 8: Diagnostic guidelines used [file 12884_2025_8472_MOESM8_ESM.png]
