# Supplementary material for: Variation in gestational diabetes diagnosis and care practices in maternity services in three high-income countries; a cross-sectional survey
Source: BMC Pregnancy Childbirth. 2025 Dec 6;26:165. doi: 10.1186/s12884-025-08472-5 (PMC12908269; doi:10.1186/s12884-025-08472-5)
Supplement: Supplementary file 7 — Supplementary Material 7. Supplementary file 7: BMI thresholds for GDM screening [file 12884_2025_8472_MOESM7_ESM.docx]

**Supplementary file 7:** BMI thresholds used for GDM screening in early (12-16 weeks) and later (24-28 weeks) pregnancy.

BMI thresholds for early pregnancy screening

|  | **Australia** | **England & Wales** | **Ireland** |
| --- | --- | --- | --- |
|  | **N=12** | **N=12** | **N=6** |
| **BMI>25kg/m^2^** | 2 (16.7%) | 0 (0.0%) | 1 (16.7%) |
| **BMI>30kg/m^2^** | 7 (58.3%) | 9 (75.0%) | 4 (66.7%) |
| **BMI >35kg/m^2^** | 3 (25.0%) | 1 (8.3%) | 1 (16.7%) |
| **BMI >40kg/m^2^** | 2 (16.7%) | 1 (8.3%) | 1 (16.7%) |
| **Other** | 0 (0.0%) | 3 (25.0%) | 0 (0.0%) |

*values in column add to more than 100% where individuals from the same centre gave differing responses.

BMI thresholds for later pregnancy screening

|  | **Australia** | **England & Wales** | **Ireland** |
| --- | --- | --- | --- |
|  | **N=3** | **N=32** | **N=10** |
| **BMI>25kg/m^2^** | 1 (33.3%) | 0 (0.0%) | 1 (10.0%) |
| **BMI>30kg/m^2^** | 1 (33.3%) | 28 (87.5%) | 9 (90.0%) |
| **BMI >35kg/m^2^** | 0 (0.0%) | 5 (15.6%) | 3 (30.0%) |
| **BMI >40kg/m^2^** | 0 (0.0%) | 0 (0.0%) | 1 (10.0%) |
| **Other** | 1 (33.3%) | 0 (0.0%) | 0 (0.0%) |

*values in column add to more than 100% where individuals from the same centre gave differing responses.
