# Supplementary material for: Variation in gestational diabetes diagnosis and care practices in maternity services in three high-income countries; a cross-sectional survey
Source: BMC Pregnancy Childbirth. 2025 Dec 6;26:165. doi: 10.1186/s12884-025-08472-5 (PMC12908269; doi:10.1186/s12884-025-08472-5)
Supplement: Supplementary file 9 — Supplementary Material 9. Supplementary file 9: Care offered to women diagnosed with GDM. [file 12884_2025_8472_MOESM9_ESM.docx]

**Supplementary file 9:** Care offered to women diagnosed with GDM

| GDM care offered (n=43) | N = 12 (%) Australia | N = 15 (%) Ireland | N = 26 (%) UK |
| --- | --- | --- | --- |
| *Dietary advice* | *12 (100%)* | *15 (100%)* | *25 (96%)* |
| *Physical activity advice* | *12 (100%)* | *15 (100%)* | *20 (77%)* |
| *Blood glucose testing training and support* | *12 (100%)* | *15 (100%)* | *25 (96%)* |
| *Medication* | *11 (92%)* | *15 (100%)* | *24 (92%)* |
| *Additional growth scans* | *12 (100%)* | *14 (93%)* | *26 (100%)* |
